# Supplementary material for: Adherence to iron-folic acid supplementation and its associated factors among pregnant women in Kenya: A multilevel data analysis of the 2022 Kenyan Demographic and Health Survey
Source: PLoS One. 2025 Aug 1;20(8):e0329458. doi: 10.1371/journal.pone.0329458 (PMC12316296; doi:10.1371/journal.pone.0329458)
Supplement: S1 Table — (DOCX) [file pone.0329458.s001.docx]

**S1 Table: Lists of individual and community level factors affecting adherence to iron-folic acid supplementation among pregnant women in Kenya, KDHS 2022.**

| **Individual-level variables** | **Descriptions** |
| --- | --- |
| Maternal age (Years) | 15-19*, 20-24, 25-29, 30-34, 35-39, 40-49 |
| Sex of the household head | Male*, Female |
| Marital status of the mother | Never married*, Married, Separated |
| Maternal occupation | Employed, Unemployed* |
| Perception of the distance to HF | Not a big problem*, A big problem |
| Family size | 1-4 members*, 5-9 members, > 10 members |
| Parity (total children ever born) | Primiparous*, Multiparous |
| Currently pregnant | Yes, No* |
| ANC visits | 1-3*, > 4 |
| Timing of first ANC visit | First trimester*, Second trimester, Third trimester |
| Prenatal nurse/midwife | Yes, No* |
| **Community-level variables** |  |
| Residence | Urban*, Rural |
| Community wealth index | Low, High* |
| Community Education | Low, High* |
| Community media exposure | Low, High* |
|  | *Reference category |
