# Supplementary material for: Adherence to iron-folic acid supplementation and its associated factors among pregnant women in Kenya: A multilevel data analysis of the 2022 Kenyan Demographic and Health Survey
Source: PLoS One. 2025 Aug 1;20(8):e0329458. doi: 10.1371/journal.pone.0329458 (PMC12316296; doi:10.1371/journal.pone.0329458)
Supplement: Attachment — Submitted filename: Review comments_Adherence to iron-folic acid supplementation.pdf [file pone.0329458.s002.pdf]

Manuscript: PONE-D-24-57968

Title: Adherence to iron-folic acid supplementation and its associated factors among pregnant women in Kenya: a multilevel data analysis of 2022 Kenyan national population based survey

Review comments

Firstly, thank you very much for providing the opportunity to review this manuscript. Second, I will list out all the important comments to be incorporated within the manuscript for a better research output.

ABSTRACT

1. Page 2: line 40-41. A sample of 8460 weighted study participants ~~was~~ used in the analysis. Change the word instead of was to were.
2. Page 2: line 55-61. I can't saw a difference in the authors result and conclusion writing. It seems a duplication. Conclude your results based on the AOR findings. If AOR > 1, it shows that the factors have positive associations with your dependent variable where as If AOR < 1, it shows that the factors have negative associations with your dependent variable (protective effect).
3. Page 2: line 62-63. Arrange the keywords in terms of relevance such as Adherence, Iron-folic acid supplementation, Associated factors, pregnant women, KDHS 2022, Kenya.

Introduction

4. Page 3-6: Almost all the introduction is talked about the physiological need of Iron folic acid supplementation and some explanation about the factors related to Iron folic acid supplementation adherence. So, the authors should critically revise the introduction based on the research objective. I expect more explanations related to the adherence to Iron folic acid supplementation and associated factors among pregnant women. So, add the prevalence of adherence to Iron folic acid supplementation starting from worldwide to the local levels and factors associated to it, and condense the physiology to get more space for explanation.
5. Page 6: line 128-130: Add your reference.
6. Page 6: line 130: Therefore, our ~~goal~~ was to determine…change the word goal to formal word objective

   Methods

   Study design, setting and periods

7. The authors did not clearly state about the study setting periods rather than overstate about the KDHS. Please condense the KDHS statement and add the following points. State about the country Kenya…Where is Kenya? What about the population in the country? How many states/regions does Kenya have? State about the health infrastructure in the country…Where was the study conducted? Which states? When was the study conducted? These comments should be addressed.
8. Page 8: line 159-162: Move this paragraph to the study setting.
9. Page 8: line 165-167: 8460 pregnant women who had received iron-folate supplements were included in this study, and it was inquired of them how many days they had taken iron tablets or syrups during <u>their previous pregnancy</u> (**Fig 1**). What was the period in which their previous pregnancies occurred? What do you think about recall biases if one woman has last pregnancy at age of 18 years and the other at age of 38 years old? Who will provide more evident information about the adherence to iron-folate supplementation? If the last pregnancy had not time bound, the study subjects had provided incorrect information and your output was vulnerable to recall bias.
10. Page 8: line 172-173: Could the authors add the authorization letter from Measure DHS to access and utilize the data as a supplementary material?
11. Page 10: line 202-203: “poor wealth index” needs other operational definition. What does poor wealth index mean? How it can be calculated. Your research should be easily understandable by readers.
12. What was your target population and study population?
13. What was your exclusion criteria’s probably from your data source/KDHS ?
14. Page 13: line 282: Use the word no instead of not…had <u>no</u>~~t~~ occupation (not working).

Result

15. Page 16: line 318-319: It says “Being in age group from 20-24 years was 1.32 times more likely to adhere to iron-folic acid as compared with age group from 15-19 years [AOR = 1.32 (95% CI: 1.07, 1.63)].” Where is a p-vale? I can’t see the p-value in all associated variables both in the explanation and within the table. So, please add the p-value in order to see the significance value at least in either of the two. Note: It works for all significant variables.

Discussion

16. Page 19: line 359-364: The possible reasons should be cited from credible studies. And try to cite research evidence of why these variables are associated, not just supposition.
17. Page 19: line 359-364: Provide your reasons individually why your research finding was higher than X studies and why your research finding were lower than X studies based on relevant references/studies/citation. It works for all discussions.
18. Page 19: line 365-367: Remove duplication of paragraphs. It was already written in the result component of your manuscript.

Strengths and limitations of the study

19. Page 23: line 452-453: Overall, these findings will help to improve the nation's iron deficiency anemia counseling and intervention methods. You mentioned here is to improve the iron-folic deficiency anemia counseling and interventions. What about megaloblastic anemia, neural tube defect…prevented through iron-folic supplementation??

Annex

20. Figures in Annex have no numbers.

Overall comment

This study is well-conducted, and the researchers are commended for their work thorough evaluating magnitude of Adherence to iron-folic acid supplementation and its associated factors among pregnant women in Kenya. The study is effectively structured, with a clear identification of the impact of Adherence to iron-folic acid supplementation with well thought recommendations for public health interventions. However, to enhance its credibility and ensure clear communication of the findings, the researchers should address the minor comments and suggestions provided above. Once these revisions are made, the study will be ready for publication and will provide valuable insights into malaria prevention and control.
