# Supplementary material for: Adherence to iron-folic acid supplementation and its associated factors among pregnant women in Kenya: A multilevel data analysis of the 2022 Kenyan Demographic and Health Survey
Source: PLoS One. 2025 Aug 1;20(8):e0329458. doi: 10.1371/journal.pone.0329458 (PMC12316296; doi:10.1371/journal.pone.0329458)
Supplement: Attachment — Submitted filename: PLOS One -Iron Adherence_ Review.docx [file pone.0329458.s003.docx]

**Comments to the Authors**
Dear Authors, 
The manuscript is interesting to read. The topic is of an important public health concern.

Here are a few comments that need to be addressed:
**Introduction:**
Line 65 begins with Adherence **is** a medical context refers to….. should be revised to Adherence **as** a medical…

Line 75: Include the reference for the WHO at the end of their statement at line 76

Line 79: the ststement that begins with pregnant women should take….Please clarify whether it is a requirement. Perhaps it should read pregnant women are required to ….

Line 102: It is accompanying… should rather be it is accompanied…

Line 139: ‘The 2022 KDHS was designed…’ Please take a look at the sentence as it is not clear

**Materials and Methods**:

Line 220: Sentence on mixed effect model seems incomplete

Results:
Line 281: Regarding **to** the occupational status of the pregnant women, about 43.32% had **not** occupation – Please revise the typos in this sentence
Line 305: Get the missing reference at the end of sentence

Also reference table 2 within the text

Discussion

Please take note of grammertical errors in the work

For example check sentence on line 411
