# Supplementary material for: Adherence to iron-folic acid supplementation and its associated factors among pregnant women in Kenya: A multilevel data analysis of the 2022 Kenyan Demographic and Health Survey
Source: PLoS One. 2025 Aug 1;20(8):e0329458. doi: 10.1371/journal.pone.0329458 (PMC12316296; doi:10.1371/journal.pone.0329458)
Supplement: Attachment — Submitted filename: Response to Reviewers.docx [file pone.0329458.s005.docx]

**March 25, 2025**

**Rebuttal letter**

**To: PLOS ONE Editorial Office**

**Authors’ point-by-point Responses to a Manuscript ID: PONE-D-24-57968**

Dear Editor in Chief, Greetings.

# We have submitted these authors' responses to peer-review comments and questions for a manuscript entitled: Adherence to iron-folic acid supplementation and its associated factors among pregnant women in Kenya: a multilevel data analysis of 2022 Kenyan national population-based survey.

Dear Editor, first, we would like to thank the PLOS ONE Editorial Office members, especially the chief editor, who timely assigned the competent academic editor and facilitated the progress of the review forum. Second, our special thanks go to the academic editor for his editorial contribution and for assigning the potential skilled and experienced reviewers in the field promptly. Moreover, our deepest gratitude goes to the esteemed reviewers for their constructive comments and scientific contributions that helped us to improve the quality of this manuscript. Dear academic Editor and reviewers, we have made the necessary corrections and responses to those comments raised by academic editor and reviewers point by point, page by page, and line by line sequentially. Accordingly, we have added the authors’ responses in the review forum, as well as the revised manuscript and the tracked changes to the manuscript on the web page.

With regards!

Gebrie Getu Alemu

[gebryegetu27@gmail.com](mailto:gebryegetu27@gmail.com)

University of Gondar, Ethiopia

PO. Box 196

Corresponding Author

**Table:** **Authors’ responses to editor and peer-review of reviewers**

| **Academic Editor's comments and Authors’ responses** | |
| --- | --- |
| **Journal Requirements** | **Authors’ Responses** |
| 1. When submitting your revision, we need you to address these additional requirements. Please ensure that your manuscript meets PLOS ONE's style requirements, including those for file naming. The PLOS ONE style templates can be found at  <https://journals.plos.org/plosone/s/file?id=wjVg/PLOSOne_formatting_sample_main_body.pdf> and  [https://journals.plos.org/plosone/s/file?id=ba62/PLOSOne_formatting_sample_title_authors_affiliations.pdf](https://track.editorialmanager.com/CL0/https:%2F%2Fjournals.plos.org%2Fplosone%2Fs%2Ffile%3Fid=ba62%2FPLOSOne_formatting_sample_title_authors_affiliations.pdf/1/010f019570b34471-333156dc-b505-486c-9ad8-5b6e13bff79b-000000/niI06u9RYZozaF9zW6Rsn_g0JN9WThcCy-T6SwG5qAU=201) | Dear editor, thank you for the comment. We have checked the given link to revise the document and we used the author formatting checklist to confirm that our paper meets PLOS ONE's typesetting requirements for references, Tables, and Figures. We ensured that our manuscript meets PLOS ONE’S style including file naming. |
| 2. Please note that funding information should not appear in any section or other areas of your manuscript. We will only publish funding information present in the Funding Statement section of the online submission form. Please remove any funding-related text from the manuscript. | Dear editor, thank you for the feedback. We have removed the funding information in the revised manuscript. |
| 3. We note that your Data Availability Statement is currently as follows: “All relevant data are within the manuscript and in Supporting Information files.”  Please confirm at this time whether your submission contains all raw data required to replicate the results of your study. Authors must share the “minimal data set” for their submission. PLOS defines the minimal data set to consist of the data required to replicate all study findings reported in the article, as well as related metadata and methods  (<https://journals.plos.org/plosone/s/data-availability#loc-minimal-data-set-definition>).  For example, authors should submit the following data:  - The values behind the means, standard deviations and other measures reported;  - The values used to build graphs;  - The points extracted from images for analysis.  Authors do not need to submit their entire data set if only a portion of the data was used in the reported study.  If your submission does not contain these data, please either upload them as Supporting Information files or deposit them to a stable, public repository and provide us with the relevant URLs, DOIs, or accession numbers. For a list of recommended repositories, please see  <https://journals.plos.org/plosone/s/recommended-repositories>.  If there are ethical or legal restrictions on sharing a de-identified data set, please explain them in detail (e.g., data contain potentially sensitive information, data are owned by a third-party organization, etc.) and who has imposed them (e.g., an ethics committee). Please also provide contact information for a data access committee, ethics committee, or other institutional body to which data requests may be sent. If data are owned by a third party, please indicate how others may request data access. | Dear editor, thank you for the comment. We have no raw data to submit. But all the analyzed data (output) are included in the manuscript in terms of texts, tables and figures. We have submitted the values behind frequencies, median, interquartile range, the values used to build graphs, the points extracted from images for analysis. We said that “All relevant data are within the manuscript and in Supporting Information files” to show the analyzed data and reported in the manuscript. |
| 4. We note you have included a table to which you do not refer in the text of your manuscript. Please ensure that you refer to Table 2 in your text; if accepted, production will need this reference to link the reader to the Table. | Dear editor, thank you for the feedback. We have cited Table 2 in the revised manuscript with cross reference as (**Table 2**) in the appropriate placement. This can be seen on **page 18,** **line 363.** |
| **Academic editor comments** | **Authors’ Responses** |
| Dear Authors, Remove reference in Abstract. | Dear editor, thank you for the feedback. We have removed the reference in the abstract. |
| Pleas reorganize the paper based on reviewer comments. Please work on Abstract, Background, Methods, Results, Discussion and Conclusion section. | Dear editor, thank you for the comment. We have revised the document according to the reviewer comments. We have worked and addressed the comments raised in the abstract, background, methods, results, and discussion and conclusion section. We act based on the raised comments, feedbacks and suggestions throughout the manuscript accordingly. |
| Follow standard for reporting aOR | Dear editor, thank you for the feedback. We have used the “aOR” to report the findings consistently in the revised manuscript. |
| Figure and Table should be formatted based on PLOS One guideline and scientific standards. | Dear editor, thank you for the comment. We used the author formatting checklist to confirm that our paper meets PLOS ONE's typesetting requirements for Tables, and Figures. we have run all figures included in our submission files through the PACE tool: <https://pacev2.apexcovantage.com> |
| Provide a letter of granting permission for data usage for analysis as supplementary file. [Note: HTML markup is below. Please do not edit.] | Dear editor, thank you for the feedback. We have added the authorization letter from Measure DHS to access and utilize the data as a supplementary material (**S1_File.pdf**). This can be seen on **Page 11, line 225, and we have added it on the webpage as supplementary material.** |
| **Reviewer comments and Authors’ responses** | |
| **Reviewer 1 comments** | **Authors’ Responses** |
| This study provides relevant and high scientific value information on maternal health, focusing on socio-demographic factors that could be associated with adherence to iron and folic acid supplementation among pregnant women in Kenya. Its relevance in the context of public health is indisputable, given the direct impact these micronutrients have on maternal-fetal health. | Dear reviewer, thank you for picking the important strengths and supporting insight of the research article. We feel a great pleasure for your constructive comments and scientific contributions that helped us to improve the quality of this manuscript. The specific responses for the comments are as follows. |
| 1. **Abstract:** The authors are advised to remove the abbreviation “KDHS 2022” from the keywords section, as its inclusion does not significantly contribute to the indexing or visibility of the article and may be redundant. | Dear reviewer, thank you for your comment. We have removed “KDHS 2022” from the key words as per the recommendations. |
| 2. **Introduction:** The article adequately emphasizes the importance of investigating the factors influencing adherence to iron and folic acid supplementation, highlighting it as a critical public health issue with substantial implications for maternal-fetal well-being. However, it is suggested that the authors structure a clear research question at the end of the introduction, aligned with the study's stated objective. This will enhance the clarity and direction of the study. | Dear reviewer, thank you for the comment. We have included two research questions at the end of the introduction. These research questions stated the prevalence of adherence to iron-folic acid supplementation and its associated factors which aligned with the objective of the study. The included research questions can be seen on **Page 8, line 156-160.** |
| 3. **Method:** The methodological design is appropriate and aligned with the study's objective, supporting the validity of the results. Furthermore, the authors have provided an adequate operational definition of the independent variables, contributing to the reproducibility and understanding of the approach employed. | Dear reviewer, thank you for highlighting the key strengths and providing insightful support for the methodology. |
| 4. **Implications for Policy, Practice, and Future Research:** The authors are encouraged to restructure the section dedicated to the study's implications, developing and justifying each aspect in separate paragraphs. Specifically: 1. **Implications for Public Health Policy:** It is recommended to draft a specific paragraph addressing how the findings can inform the design or implementation of policies that promote greater adherence to iron and folic acid supplementation in contexts similar to Kenya. 2. **Implications for Healthcare Practice:** A second paragraph could focus on practical recommendations for healthcare providers, highlighting strategies to improve education, access, and adherence among pregnant women.   3. **Implications for Future Research:** A third paragraph should detail priority areas that require further exploration. This section must emphasize the knowledge gaps identified in the study and their relevance for optimizing adherence to iron and folic acid supplementation, with an emphasis on the sociocultural and economic context of Kenya.  Together, these adjustments will strengthen the study’s contribution, enhancing its clarity and utility for both the scientific community and decision-makers in the field of public health. | Dear reviewer, thank you for the comment. We have revised the Implications for Policy, Practice, and Future Research as per the recommendation. We have separately rewritten the implications with Policy, Practice, and Future Research sub-sections. The changes can be seen **Page 27-28, line 529- 550.** |
| **Reviewer 2 comments** | **Authors’ Responses** |
| **Review comments**  Firstly, thank you very much for providing the opportunity to review this manuscript. Second, I will list out all the important comments to be incorporated within the manuscript for a better research output. | Dear reviewer, thank you for the comments and suggestions. We appreciate your comments and feedbacks. We are deeply grateful for your constructive feedback and scientific contributions, which have significantly enhanced the quality of this manuscript. Below are our detailed responses to your comments. |
| **ABSTRACT**   1. Page 2: line 40-41. A sample of 8460 weighted study participants was used in the analysis. Change the word instead of was to were. | Dear reviewer, thank you for the suggestion. We have replaced as per the recommendation. The changes can be seen **Page 2, line 41.** |
| 2. Page 2: line 55-61. I can’t saw a difference in the authors result and conclusion writing. It seems duplication. Conclude your results based on the AOR findings. If AOR > 1, it shows that the factors have positive associations with your dependent variable where as If AOR < 1, it shows that the factors have negative associations with your dependent variable (protective effect). | Dear reviewer, thank you for the comment. It is a nice comment. We have tried to conclude the results based on the aOR findings as a positive and negative association separately. The changes can be seen **Page 3, line 56-64.** |
| 3. Page 2: line 62-63. Arrange the keywords in terms of relevance such as Adherence, Iron-folic acid supplementation, associated factors, pregnant women, KDHS 2022, Kenya. | Dear reviewer, thank you for the comment. We have rewritten the key words as per the recommendation. We have removed “KDHS 2022” from the key words because of its relevance of inclusion does not significantly contribute to the indexing or visibility of the article. The changes can be seen **page 4, line 65-66.** |
| **Introduction**   4. Page 3-6: Almost all the introduction is talked about the physiological need of Iron folic acid supplementation and some explanation about the factors related to Iron folic acid supplementation adherence. So, the authors should critically revise the introduction based on the research objective. I expect more explanations related to the adherence to Iron folic acid supplementation and associated factors among pregnant women. So, add the prevalence of adherence to Iron folic acid supplementation starting from worldwide to the local levels and factors associated to it, and condense the physiology to get more space for explanation. | Dear reviewer, thank you for your constructive comment. We have gotten the comment is nice and valuable. As much as possible we have summarized and added the prevalence of adherence to iron-folic acid supplementation from global to local in the revised manuscript. The changes can be seen in the introduction section of the manuscript **Page 4-5, line 82-94.** |
| 5. Page 6: line 128-130: Add your reference. | Dear reviewer, thank you for the comment. This is due to lack of evidence/source as far as our exhaustive searching. To our searching we haven’t found national representative evidence regarding IFAS in Kenya. It is our idea not others work so that we can’t acknowledge this sentence as others. |
| 6. Page 6: line 130: Therefore, our goal was to determine…change the word goal to formal word objective | Dear reviewer, thank you for the comment. We have changed as per the recommendation. The changes can be seen **Page 7, line 153** |
| **Methods**  **Study design, setting and periods**   7. The authors did not clearly state about the study setting periods rather than overstate about the KDHS. Please condense the KDHS statement and add the following points. State about the country Kenya…Where is Kenya? What about the population in the country? How many states/regions does Kenya have? State about the health infrastructure in the country…Where was the study conducted? Which states? When was the study conducted? These comments should be addressed. | Dear reviewer, thank you for your valuable insight. The comments are nice and constrictive. We have addressed all the comments. We have Stated about the country Kenya…Where it is found, about the population in the country, the states/regions, the health infrastructure in the country, where the study was conducted, the states in which the study was conducted, and time when the study was conducted. This all can be seen in **Page 8-9, lines 163-187. Kindly see them.** |
| 8. Page 8: line 159-162: Move this paragraph to the study setting. | Dear reviewer, thank you for the suggestion. We have taken the paragraph into the study setting. This can be seen **Page 8, lines 168-173.** |
| 9. Page 8: line 165-167: 8460 pregnant women who had received iron-folate supplements were included in this study, and it was inquired of them how many days they had taken iron tablets or syrups during their previous pregnancy (Fig 1). What was the period in which their previous pregnancies occurred? What do you think about recall biases if one woman has last pregnancy at age of 18 years and the other at age of 38 years old? Who will provide more evident information about the adherence to iron-folate supplementation? If the last pregnancy had not time bound, the study subjects had provided incorrect information and your output was vulnerable to recall bias. | Dear reviewer, thank you for the comment. Women with no birth in the past five years preceding the survey were excluded from the study. Because from the beginning the data collectors first asked the woman “did you give birth in the last five years”? If the answer was “Yes” then they proceeded to the next questionnaire. If the answer was “No” then the woman was excluded and they didn’t further ask the questionnaire.  The study population consisted of Kenyan women aged 15 to 49 years, who had a live birth in the five years preceding the KDHS 2022 survey I.e. between 2017 and, 2022.  Depending on criteria including life experience, cognitive load, and health literacy, women who had their previous pregnancy at 18 and 38 years old may have different accuracy of maternal recall for iron-folate supplementation adherence.  **Younger mothers (age 18)**  **Lower heath Literacy:** Because younger mothers are often less experienced with prenatal care procedures, they may not fully understand supplementation schedules and have poor recall accuracy.  **Higher cognitive load:** Balancing pregnancy with education, work and social pressure may lead to more forgetfulness or fragmented memory.  **Adherence pattern:** According to different studies including our study finding, younger women are less likely to adhere to which could correlate with underreporting potentially increasing recall bias.  **Older mothers (age 38)**  **Experience and health literacy:** Recall precision may be improved by older mothers' greater health literacy from past pregnancies or life experiences.  **Adherence pattern:** Supplementation adherence is more likely to be maintained by older women, which may lessen recall bias associated with non-adherence.  **Overall:** Older mothers (38 years old) have better adherence rates, more health literacy, and possibly more involvement in prenatal care, are more likely to give correct information about supplements adherence I.e. have reduced recall bias.  Really, there would be a recall bias. We have acknowledged the potential recall biases as a limitation. This can be seen on **Page 26, lines 508-512.** **N.B:** information pertaining to the most recent pregnancy was used to minimize recall bias. Furthermore, the DHS utilized structured interview (standardized questionnaires) which can mitigate the recall bias. |
| 10. Page 8: line 172-173: Could the authors add the authorization letter from Measure DHS to access and utilize the data as a supplementary material? | Dear reviewer, thank you for the feedback. We have added the authorization letter from Measure DHS to access and utilize the data as a supplementary material (**S1_File.pdf**). This can be seen on **Page 11, line 225, and we have added it on the webpage as supplementary material.** |
| 11. Page 10: line 202-203: “poor wealth index” needs other operational definition. What does poor wealth index mean? How it can be calculated. Your research should be easily understandable by readers. | Dear reviewer, thank you for your valuable feedback. We have taken the comment. Really it was unclear. We have used poor wealth index to express community wealth index. It is not representing community wealth index. So, we have changed poor wealth index by community wealth index. We have revised the operational definition of community wealth index and we tried to write clearly and precisely as follows.  We have operationalized community wealth index based on a household wealth index. Women were categorized by community according to whether they lived in poor or rich households. The percentage of women in each community belonging to either of these categories was then calculated. Communities were then classified as having “low” (less than 45.58%) or “high” (45.58% or greater) levels, using the median percentage (45.58%) as the cutoff point. We have used median because the nature the data was skewed. This revised operational definition is included in the revised manuscript. It can be seen on **Page 12, lines 253-257.** |
| 12. What was your target population and study population? | Dear reviewer, thank you for the question. The source and study population were as follows:  **Source population**  All Kenyan women aged 15 to 49 years, who had a live birth in the five-year preceding 2022 KDHS survey  **Study population**  The study population consisted of Kenyan women aged 15 to 49 years, who had a live birth in the five-year preceding 2022 KDHS survey between 2017 and 2022. Women who attended at least one ANC visit were included, and only information pertaining to the most recent pregnancy was used. We have included this in the revised manuscript at the methods section. It can be seen on **Page 10, line 212-220**. |
| 13. What was your exclusion criteria’s probably from your data source/KDHS? | Dear reviewer, thank you for the question. The exclusion criteria from data source/KDHS were women who were not taken iron-folic acid supplementation. |
| 14. Page 13: line 282: Use the word no instead of not…had not occupation (not working). | Dear reviewer, thank you for the feedback. We have changed “no” instead of “not” as per the recommendation. The changes can be seen **Page 16, line 337.** |
| **Result**   15. Page 16: line 318-319: It says “Being in age group from 20-24 years was 1.32 times more likely to adhere to iron-folic acid as compared with age group from 15-19 years [AOR = 1.32 (95% CI: 1.07, 1.63)].” Where is a p-vale? I can’t see the p-value in all associated variables both in the explanation and within the table. So, please add the p-value in order to see the significance value at least in either of the two. Note: It works for all significant variables. | Dear reviewer, thank you for the comment. We have included the **P-values for all variables in the table**. **Kindly** **see in Table 3*.***  ***Note: The included P-values are for Model IV (the best model among all in which we have interpreted its output).*** |
| **Discussion**   16. Page 19: line 359-364: The possible reasons should be cited from credible studies. And try to cite research evidence of why these variables are associated, not just supposition. | Dear reviewer, thank you for the comment. We have added more reasons for the possible variations (discrepancy). We have also cited the reference we have used for the possible justification of the difference. This can be seen **Page 22, Lines 405-421**. We have also tried to cite the possible reasons from credible studies for all findings (The outcome variable and significant variables which were significantly associated with the outcome variable). This can be seen in the overall discussion part of the revised manuscript. The uncited sentences are our ideas. We have used the uncited ideas to specifically highlight the implications of the study related with the significant variable to the stakeholders. |
| 17. Page 19: line 359-364: Provide your reasons individually why your research finding was higher than X studies and why your research finding were lower than X studies based on relevant references/studies/citation. It works for all discussions. | Dear reviewer, thank you for the comment. **We have discussed the reasons individually** why our research finding was higher than other studies and why lower than other studies conducted elsewhere by citing the relevant studies (references). This can be seen **Page 22, Lines 405-421**. We have also tried to discuss the significant factors associated with adherence to iron-folic acid supplementation among pregnant women in Kenya with justifications why these variables were significantly associated with it by citing the appropriate references. This can be seen **in all over parts of the discussion.** |
| 18. Page 19: line 365-367: Remove duplication of paragraphs. It was already written in the result component of your manuscript. | Dear reviewer, thank you for the comment. We have removed as per the recommendation. |
| **Strengths and limitations of the study**   19. Page 23: line 452-453: Overall, these findings will help to improve the nation's iron deficiency anemia counseling and intervention methods. You mentioned here is to improve the iron-folic deficiency anemia counseling and interventions. What about megaloblastic anemia, neural tube defect…prevented through iron-folic supplementation?? | Dear reviewer, thank you for the comment. We appreciate your comment. The comment is conducive and constructive. Really megaloblastic anemia and neural tube defects are prevented through iron-folic acid supplementation. The relationship between megaloblastic anemia and iron-folic acid supplementation is significant, particularly in preventing and treating this condition. Iron-folic acid supplementation is beneficial in reducing the incidence of megaloblastic anemia, particularly in pregnant women, by addressing folate deficiency. Moreover, iron-folic supplementation prevents Neural Tube Defect (NTD). Iron is often combined with folic acid in supplements to prevent maternal anemia, but the folic acid component directly mitigates NTD risk by ensuring sufficient red blood cell folate levels. Dear reviewer, we have tried to address the mechanisms in which how iron-folic acid supplements prevents megaloblastic anemia and neural tube defects in the **introduction section page 4, line** **75-81,** **in the strength and limitation section Page 26, line 506-508,** **and in the implications section Page 28 line 544-546. Kindly see these parts**. |
| **Annex**   20. Figures in Annex have no numbers. | Dear reviewer, thank you for the comment. Figures have numbers both in the manuscript and on the webpage. We have cited and named the figures according to the journal guideline (requirement). |
| **Overall comment**  This study is well-conducted, and the researchers are commended for their work thorough evaluating magnitude of Adherence to iron-folic acid supplementation and its associated factors among pregnant women in Kenya. The study is effectively structured, with a clear identification of the impact of Adherence to iron-folic acid supplementation with well thought recommendations for public health interventions. However, to enhance its credibility and ensure clear communication of the findings, the researchers should address the minor comments and suggestions provided above. Once these revisions are made, the study will be ready for publication and will provide valuable insights into malaria prevention and control. | Dear reviewer, thank you for picking the important strengths and supporting insight of the research article. We feel a great pleasure for your constructive comments and scientific contributions that helped us to improve the quality of this manuscript. We have addressed and given specific responses for the raised comments and suggestions. |
| **Reviewer 3 comments** | **Authors’ Responses** |
| Dear Authors,  The manuscript is interesting to read. The topic is of an important public health concern.  Here are a few comments that need to be addressed: | Dear reviewer, thank you for identifying the significant strengths and providing insightful support for the research article. We feel a great pleasure for your constructive comments and scientific contributions that helped us to improve the quality of this manuscript. The specific responses for the comments are as follows. |
| **Introduction:** Line 65 begins with Adherence **is** a medical context refers to….. Should be revised to Adherence **as** a medical…  Line 75: Include the reference for the WHO at the end of their statement at line 76  Line 79: the statement that begins with pregnant women should take….Please clarify whether it is a requirement. Perhaps it should read pregnant women are required to ….  Line 102: It is accompanying… should rather be it is accompanied…  Line 139: ‘The 2022 KDHS was designed…’ Please take a look at the sentence as it is not clear | Dear reviewer, thank you for the feedback. We have changed “as” instead of “is” as per the recommendation. The changes can be seen **Page 4, line 68**.  Dear reviewer, thank you for the comment. We have included the reference for the WHO at the end of their statement. This can be seen **Page 5, line 99**.  Dear reviewer, thank you for the comment. We have rephrased the sentence. We have changed the statement “pregnant women should” by “pregnant women are required to” as per the suggestion. The changes can be seen **Page 5, line 102.**  Dear reviewer, thank you for the feedback. We have changed “accompanying…” By “accompanied…” as per the recommendation. The changes can be seen **Page 6, line 125.**  Dear reviewer, thank you for the insight. We have rephrased the sentence to be clear. **Kindly see** **Page 9, Line 184-187**. |
| **Materials and Methods**:  Line 220: Sentence on mixed effect model seems incomplete | Dear reviewer, thank you for the comment. We have completed the incomplete sentence as “This implies that the necessity of using a multilevel mixed effect logistic regression model to account for the variability between clusters”. The changes can be seen on **Page 13, Line 275-277.** |
| **Results:** Line 281: Regarding to the occupational status of the pregnant women, about 43.32% had **not** occupation – Please revise the typos in this sentence  Line 305: Get the missing reference at the end of sentence  Also reference table 2 within the text | Dear reviewer, thank you for the feedback. We have changed “no” instead of “not” to make sense full and grammatically correct. The changes can be seen **Page 16, line 337.**  Dear reviewer, thank you for the feedback. We have gotten the missing reference at the end of sentence and we have removed it. We have cited Table 2 in the revised manuscript with cross reference as (**Table 2**) in the appropriate placement. This can be seen on **page 18,** **line 363.** |
| **Discussion**  Please take note of grammatical errors in the work  For example, check sentence on line 411 | Dear reviewer, thank you for the comment. We have rewritten the sentence to be sense full and grammatically correct. This can be seen **Page 24, Line 468-470**. We have carefully checked and corrected the grammatical errors, punctuations and spelling mistakes of the overall manuscript. We have also applied the Grammarly tool to improve the overall language quality of the document. |
