# Supplementary material for: Adherence to iron-folic acid supplementation and its associated factors among pregnant women in Kenya: A multilevel data analysis of the 2022 Kenyan Demographic and Health Survey
Source: PLoS One. 2025 Aug 1;20(8):e0329458. doi: 10.1371/journal.pone.0329458 (PMC12316296; doi:10.1371/journal.pone.0329458)
Supplement: Attachment — Submitted filename: Response_to_Reviewers_auresp_2.docx [file pone.0329458.s006.docx]

**May 05, 2025**

**Rebuttal letter**

**To: PLOS ONE Editorial Office**

**Authors’ point-by-point Responses to a Manuscript ID: PONE-D-24-57968R1**

Dear Editor in Chief, Greetings.

We have submitted these authors' responses to peer-review comments and questions for a manuscript entitled: Adherence to iron-folic acid supplementation and its associated factors among pregnant women in Kenya: a multilevel data analysis of the 2022 Kenyan Demographic and health Survey

# Dear Editor, first, we would like to thank the PLOS ONE Editorial Office members, especially the chief editor, who timely assigned the competent academic editor and facilitated the progress of the review forum. Second, our special thanks go to the academic editor for his editorial contribution and for assigning the potential skilled and experienced reviewers in the field promptly. Moreover, our deepest gratitude goes to the esteemed reviewers for their constructive comments and scientific contributions that helped us to improve the quality of this manuscript. Dear academic Editor, we have made the necessary corrections and responses to those additional comments raised by the editors point by point, page by page, and line by line sequentially. Accordingly, we have added the authors’ responses in the review forum, as well as the revised manuscript and the tracked changes to the manuscript on the web page.

With kind regards!

Gebrie Getu Alemu

[gebryegetu27@gmail.com](mailto:gebryegetu27@gmail.com)

University of Gondar, Ethiopia

PO. Box 196

Corresponding Author

**Table:** **Authors’ responses to editors**

| **Academic Editor's comments and Authors’ responses** | |
| --- | --- |
| **Academic Editor's comments** | **Authors’ Responses** |
| 1. I have some concern in the manuscript. I enjoyed reading your manuscript which still requires revision. I have included my comments below. I suggest reworking in almost in all the section. Remove unnecessary wording, try to make it catchy with important information. Provide an opportunity to proofread to your colleagues before submission which may help to reduce unnecessary words. | Dear Editor, thank you for the comments and suggestions. We appreciate your comments and feedbacks. We are deeply grateful for your constructive feedback and scientific contributions, which have significantly enhanced the quality of this manuscript. Below are our detailed responses to your comments. We have tried to rework in all sections of the manuscript. We have removed the unnecessary wording; we try to make the overall document catchy with the important information. All the authors of the manuscript read and make the necessary corrections in all sections of the manuscript. |
| 2. Please submit your revised manuscript by Jun 04 2025 11:59PM. If you will need more time than this to complete your revisions, please reply to this message or contact the journal office at [plosone@plos.org](mailto:plosone@plos.org). | Dear editor, thank you for the feedback. We have submitted the revised manuscript with in the given time frame. |
| 3. I suggest to make your abstract catchy, don’t mention all the categories of the age. Try to mention one or main highlight about the categories of age. | Dear editor, thank you for the suggestion. We have removed the unnecessary wordings in the abstract. We make the abstract catchy with the important information. The changes can be seen **page 2-3, lines 32-60 in the revised manuscript.**  We have rewritten and rephrased the categories of age with the main highlight. The changes can be seen **page 3, line 46-47 in the revised manuscript.** |
| 4. We don’t need a separate research question. Therefore, please incorporate in the last paragraph of the introduction section. | Dear editor, thank you for the feedback. We have removed the research question written separately. We have incorporated it in the last paragraph of the introduction section. This can be seen page **7, lines 145-147.** |
| 5. Why not to separate into three different sections for this? - Study setting, design and periods. | Dear editor, thank you for the comment. We have separately rewritten the section into study setting, study design and study period subtitles. The changes can be seen **pages 7-8, lines 149-167 in the revised manuscript. Kindly see these sections.** |
| 6. Source population- it is better to mention the number of the source population.  How many women delivered in the year 2022 and provide the relevant citation. Line 218- cite the relevant reference. | - Dear editor, thank you for the comment and question. We have mentioned the number of live births in the source population. This can be seen **Page 10, lines 202-204.** - We have also mentioned in the data source and sampling procedure clearly and briefly. This can be seen **Page 9, lines 191-198.** We have also summarized it in **Fig 1**. Kindly see these in the revised manuscript. - According to the 2022 Kenya Demographic and Health Survey (KDHS), 11,863 **women reported a birth in the last 5 years.** Of these, 9,312 women had at least one ANC visit and were the study population for our study. |
| 7. Ethical approval and consent to participate- Please reorganize the section. Mention about the details how the ethical consideration was done, how informed consent was taken, how the interview was done? Please read the KDHS once again for details. | Dear editor, thank you for the feedback. We have reorganized and mentioned about the details of the ethical consideration and the informed consent. This can be seen **page 10, lines 211-221 in the revised manuscript.**  We have also stated how the interview was done. This can be seen **page 10, lines 188-191. Kindly see these sections.** |
| 8. Line 216-265. Please include all the information in a table a supplementary file. Just brief about the variables you have considered for analysis in a paragraph. | Dear editor, thank you for the comment. We have summarized and included all the variables in a table. We uploaded this table as a supplementary material named as **S1_Table on the webpage as supplementary material.**  We have tried to rewrite the variables we have used for analysis shortly and precisely. |
| 9. Incorporate the (Data management and analysis) into statistical analysis section. | Dear editor, thank you for the suggestion. We have added the data management and analysis part into statistical analysis section. The changes can be seen **page 12, lines 253-256.** |
| 10. Results-  Table 1. Please take care of elements mention? Maternal age should mention in years, right? Maternal age (Years)  Also, how to mention 45-49 (73) and 0.86? – I think it is better to incorporate into previous category.  It is important to create a subsection for- Prevalence of adherence to iron-folic acid supplementation among 350 pregnant women in Kenya. I found the result was mention in only a line. I think it is better not to mention a separate section.  Present your findings in an analytical way. | Dear editor, thank you for the feedback. We have mentioned maternal age in years. The correction as per the recommendation can be seen **Page 15, Table 1**  Dear editor, thank you for the comment. We have merged the age category of 45-49 and 40-44 years into the age category of 40-49 years. So the age category of 45-49 years was incorporated in the age category 40-49 with the age category of 40-44 years. The changes can be seen **Page 15, Table 1.**  Dear editor, thank you for the feedback. We have removed the subtitles “Prevalence of adherence to iron-folic acid supplementation among pregnant women in Kenya” in the revised manuscript.  Dear editor, thank you for the feedback. We have presented the findings in an analytical way. |
| 11. Discussion.  Line 397-404- Please highlight your findings, what is your major finding of your study. Please review relevant literature to organize your section. | Dear reviewer, thank you for the comment. We have highlighted the major finding of the study. We have reviewed the literatures to rewrite the discussion. |
| 12. You need to compare and contrast your result. Please compare and contrast your result and provide the reason why it is so? – Why it is similar to Ethiopia? | Dear Editor, thank you for the comment and question. We tried to compare and contrast the findings in this study with other relevant studies. We have added more reasons for the possible similarities and variations (discrepancy) of our study with others. We have also cited the reference used for the possible reason (justification) of the similarities and differences. This can be seen **Page 20-24, Lines 374-463**. We have discussed why it was similar with Ethiopia. This can be seen **Page 20, Lines 377-384.** We have also tried to cite the possible reasons from credible studies for all findings (The outcome variable and significant variables which were significantly associated with the outcome variable). This can be seen in the overall discussion part of the revised manuscript. The uncited sentences are our ideas. We have used the uncited ideas to specifically highlight the implications of the study related with the findings to the stakeholders. |
| 13. In your example- Line 404-406, how it is similar to WHO recommendation, what does WHO talks about IFAS? | Dear Editor, thank you for the question. We have found suboptimal IFAS adherence among pregnant women in Kenya when compared with WHO guidelines. Our study found that only 61.72% of mothers in Kenya took IFAS according to WHO recommendations (Taking iron tablets or syrups for ≥ 90 days during antenatal care). This means that 61.72% of pregnant women in Kenya took IFAS similarly with the WHO recommendation.  **Generally, WHO talks about IFAS as follows:**  The WHO recommends daily iron and folic acid supplementation (IFAS) for all pregnant women, regardless of their anemia status, especially in countries where anemia prevalence among pregnant women is 40% or higher. The standard WHO guideline advises a combined tablet containing 60 mg of elemental iron and 400 µg (0.4 mg) of folic acid throughout pregnancy to reduce the risk of maternal anemia, iron deficiency, low birth weight, and neural tube defects in newborns. Adherence to iron-folic acid supplementation (IFAS) was defined as taking iron tablets or syrups for ≥ 90 days during antenatal care. We have discussed in detail how WHO talks about IFAS in the introduction section of the manuscript. |
| 14. The discussion section is loosely written. Please rewrite the whole section, reviewing similar literature published in PLOS One. I found the references are not organized as per the standard; please follow the standard guideline of PLOS One. See the full reference in the link-  <https://pubmed.ncbi.nlm.nih.gov/31777771/>  What is SSA in the reference 12 in the text in the discussion section, I need to read the reference list to understand it. Please mention the full form whenever necessary? | Dear Editor, thank you for the feedback and question. We tried to revised and rewrite the whole parts of the discussion as per the recommendation.  We have also carefully seen all the references and we found some references were not as per the standard. We have made modification for some reference and we have removed some of them and replaced with other appropriate and standard references.  We have referred the given link via  <https://pubmed.ncbi.nlm.nih.gov/31777771/> and we make our best to align with the standard.  Dear editor, previously we had cited this reference (reference 12) from Google Scholar with the website of  <https://scholar.google.com/scholar?hl=en&as_sdt=0%2C5&q=Adherence+to+iron+supplementation+in+22+subSaharan+African+countries+and+associated+factors+among+pregnant+women%3A+a+large+population-based+study&btnG>=  We had downloaded it from the Google Scholar and cite automatically. But now we have cited it from **PubMed** with the link <https://pubmed.ncbi.nlm.nih.gov/31777771/>  The full form of the current reference is here  Ba DM, Ssentongo P, Kjerulff KH, Na M, Liu G, Gao X, et al. Adherence to Iron Supplementation in 22 Sub-Saharan African Countries and Associated Factors among Pregnant Women: A Large Population-Based Study. Curr Dev Nutr. 2019;3(12):nzz120. Epub 20191021. doi: 10.1093/cdn/nzz120. PubMed PMID: 31777771; PubMed Central PMCID: PMCPMC6867960.  We believed that it is as per the standard. Kindly see with the link <https://pubmed.ncbi.nlm.nih.gov/31777771/> |
| **Strengths and limitations of the study-**  15. Separate into two paragraphs. What are the strengths, policy relevance, global relevance, developing countries relevancy, why it is important, what is the extra that this report explored rather than DHS report?  16. What are the limitations? | Dear editor, thank you for the comment. We have separated the strengths and limitations paragraph into to two. The first paragraph states about the strengths of the study. We have incorporated the implications of the study which deals with policy relevance, global relevance, developing countries relevance, importance, the extra that this report explored rather than DHS report in the strength of the study. This can be seen **Page 24-25, Lines 465-482.**  Similarly the second paragraph states about the limitations of the study. Here we also deal the implication of the study for researchers in the future. This can be seen **Page 25, Lines 483-491** |
| **Conclusion-**  17. Write your conclusion based on your results, some of the information about WHO recommendation need to go to the discussion section. | Dear editor, thank you for the feedback. We have rewritten the conclusion based on the findings. Kindly see **Page 25, Lines 493-496.** We have taken the information about WHO recommendation into the discussion section. |
| 18. I found this section is very important- Implications for policy, practice and future research. However, I suggest limiting your words and incorporating into the strengths and limitation section. | Dear editor, thank you for identifying the significant strengths and providing insightful support for the implication. We have condensed the lengthy words and incorporated into the strengths and limitations section accordingly. |
| 19. Figure 2. Is it important to mention outcome status in the figure? Please take care of your color used? Make the figure standard; display the number and percentage together. Legend should mention in standard way, why to include black background here? | Dear editor, thank you for the feedback. We have modified **Figure 2**. We have removed outcome status in the figure. We have made the figure as per the standard and the number and percentage displayed together. We have removed the black color in the background and we made the legend standard. Additionally, we have run all the figures on <https://pacev2.apexcovantage.com> to report as per the standard. |
| 20. I found references are not accurately organized in reference list. Please take care while citing and referring. See reference 16- MoH Kenya. National Iron & Folic Acid Supplementation, where can we read the document? | Dear Editor, thank you for your constructive comment. We have gotten the comment is nice and valuable. We have revised the references. We have correctly cited the given reference asked here in the revised manuscript as  “Ministry of Health RoK. National Iron and Folic Acid Supplementation Communication Strategy, Nairobi, Kenya: Government of Kenya. 2013”  <https://www.advancingnutrition.org/sites/default/files/2021-03/tagged_ifas_national_comunication_strategy_2013-2017.pdf>  This can be seen in the reference number 24 in the revised manuscript.  The document is available in the link given here. Kindly see  <https://www.advancingnutrition.org/sites/default/files/2021-03/tagged_ifas_national_comunication_strategy_2013-2017.pdf>  We have made the necessary modifications for all references and report as per the standards. |
| 21. To cite KDHS, you need to use the given reference in the report. Please read the report once again. I found the given citation for KDHS is- KNBS and ICF. 2023. Kenya Demographic and Health Survey 2022. Key Indicators Report. Nairobi, Kenya, and Rockville, Maryland, USA: KNBS and ICF. | Dear Editor, thank you for the suggestion. We have revised and correctly cited the reference.  Here is the corrected reference  KNBS, ICF. Kenya Demographic and Health Survey 2022 Key Indicators Report, Nairobi, Kenya, and Rockville, Maryland, USA: KNBS and ICF. <https://dhsprogram.com/pubs/pdf/PR143/PR143.pdf> 2023. This can be seen in the reference number 34 in the revised manuscript. |
| 22. While revising your submission, please upload your figure files to the Preflight Analysis and Conversion Engine (PACE) digital diagnostic tool, [https://pacev2.apexcovantage.com/](https://track.editorialmanager.com/CL0/https:%2F%2Fpacev2.apexcovantage.com%2F/1/010f019656163c19-8666203b-6b10-46e9-96d9-8f4a19883f8d-000000/Cp8aMAtivlS4-hAxKFbHCfhZ0NVPUuYYbjPbh_GqHUg=207). PACE helps ensure that figures meet PLOS requirements. | Dear editor, thank you for the comment. We used the author formatting checklist to confirm that our paper meets PLOS ONE's typesetting requirements for Figures. we have run all figures included in our submission files through the PACE tool: <https://pacev2.apexcovantage.com> |
